# Aucubin Alleviates Ulcerative Colitis in Mice by Repairing the Intestinal Barrier and Modulating the Microbiota‐Metabolism Axis

**DOI:** 10.1002/fsn3.72436

**Published:** 2026-10-15

**Authors:** Yanghui Bi, Pengyu Chen, Shuya Jia, Shiyan Jia, Zhanhao Lian, Yuanfang Zheng, Jiabin Guo, Jianxia Xue, Sinan Cheng

**Affiliations:** ^1^ School of Basic Medical Sciences Changzhi Medical College Changzhi Shanxi China; ^2^ Shanxi Provincial Center for Upper Gastrointestinal Cancer Research and Clinical Translation Changzhi Medical College Changzhi Shanxi China; ^3^ Four “Batches” Innovation Project of Invigorating Medical Through Science and Technology of Shanxi Province‐Key Laboratory of Esophageal Cancer Basic Research and Clinical Transformation Heping Hospital Affiliated to Changzhi Medical College Changzhi Shanxi China; ^4^ Department of Obstetrics Heping Hospital Affiliated to Changzhi Medical College Changzhi Shanxi China

**Keywords:** aucubin, gut barrier, intestinal barrier, tryptophan metabolism, ulcerative colitis

## Abstract

Ulcerative colitis (UC) is a chronic inflammatory bowel disease with a complex pathogenesis, necessitating the development of safe and effective therapeutic interventions. This study aimed to investigate the protective effects and underlying mechanisms of Aucubin (Auc) against dextran sulfate sodium (DSS)‐induced UC in mice. Using a DSS‐induced colitis model, we systematically evaluated the multifaceted effects of Auc by assessing the Disease Activity Index (DAI), colon length, histopathological changes, immunohistochemical staining, ELISA results, proteomic profiles, 16S rRNA sequencing data, and untargeted metabolomic analyses. Compared with the DSS group, Aucubin high‐dose (Auc‐H, 100 mg/kg) treatment significantly attenuated DSS‐induced body weight loss, DAI score elevation, and colon shortening (all *p* < 0.05). The therapeutic efficacy of Auc‐H was comparable to that of the positive control drug 5‐aminosalicylic acid (5‐ASA). Mechanistically, Auc restored intestinal barrier integrity by stimulating mucin 2 (Muc2) secretion and upregulating the expression of zonula occludens‐1 (ZO‐1) and Occludin, which in turn significantly reduced serum lipopolysaccharide (LPS) concentrations (*p* < 0.001). Proteomic analysis identified 2180 DSS‐induced differentially expressed proteins, of which 679 were significantly counter‐regulated by Auc‐H, mainly involving the complement and coagulation cascades and immune‐inflammatory pathways. Furthermore, gut microbiota analysis showed that Auc promoted the growth of beneficial bacteria, improving microbial balance. Untargeted metabolomics further revealed that Auc‐H significantly restored the levels of 120 DSS‐induced differential metabolites, including 86 upregulated and 34 downregulated metabolites, among which were protective tryptophan‐derived indole metabolites (*p* < 0.05). Consequently, Aucubin exhibits significant clinical potential in alleviating UC via a multifaceted mechanism. These effects include reduced inflammation, restored epithelial barrier integrity, and modulated microbial metabolism. These findings provide crucial experimental evidence supporting the potential application of Auc as a preventive therapy for UC.

## Graphical abstract

Aucubin (Auc), an iridoid glycoside, alleviates dextran sulfate sodium (DSS)‐induced ulcerative colitis (UC) in mice through a microbiota–metabolite–barrier axis. Preventive Auc (100 mg/kg) attenuates body‐weight loss, Disease Activity Index elevation and colon shortening to an extent comparable to 5‐aminosalicylic acid. It reshapes the gut microbiota (increased *Lachnospiraceae* and *Clostridiales*) and promotes tryptophan–indole metabolism (5‐HIAA, 5‐HI, I3C and indole‐3‐ethanol), while upregulating Muc2, ZO‐1 and Occludin, thereby reducing serum lipopolysaccharide translocation. Proteomic profiling reveals suppression of the complement and coagulation cascades and of NOD‐like receptor signaling, accompanied by decreased TNF‐α and IL‐6 and increased IL‐10.

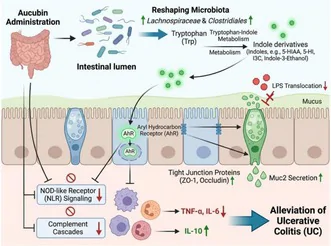

## 1 Introduction

Ulcerative colitis (UC), a chronic and often recurring inflammatory bowel disease, is characterized by persistent inflammation and ulceration of the colonic mucosa. The disease commonly manifests as abdominal pain, bloody stools, and diarrhea (Gros and Kaplan 2023). Moreover, the global prevalence of UC has risen markedly in recent years, substantially impairing the quality of life of affected patients and increasing the risk of colorectal cancer (Le Berre et al. 2023). Although several treatment options, including aminosalicylates, corticosteroids, and biologics, are available, some patients respond poorly or experience substantial side effects (Bots et al. 2018; Le Berre et al. 2020). Therefore, screening natural compounds with well‐defined efficacy and safety profiles is of great importance for the prevention and treatment of ulcerative colitis, given that the pharmacological activities and potential toxicities of natural compounds differ considerably among individual compounds and must be rigorously evaluated for each candidate.

Maintenance of gut barrier integrity is a cornerstone of intestinal homeostasis, and its disruption represents a central event in the pathogenesis of UC: a compromised mucosal barrier leads to heightened intestinal permeability and translocation of luminal pathogens, which in turn precipitates exaggerated immune responses (Orlemann et al. 2025). Growing evidence indicates that the gut microbiota and its metabolites are essential for the regulation of the gut barrier (Wu, Wei, et al. 2026; Zhang, Qiao, et al. 2025). Specifically, microbiota‐derived tryptophan metabolites, including indole and its derivatives, act as ligands for the aryl hydrocarbon receptor (AhR). AhR activation facilitates epithelial barrier repair by upregulating tight junction protein expression and modulating local anti‐inflammatory immune homeostasis (Wu, Gao, et al. 2025; Wu, Xu, et al. 2026). Conversely, indole‐producing bacteria are frequently depleted in patients with UC and in DSS‐induced colitis models, resulting in metabolic dysregulation. Targeting gut microbial metabolism therefore represents a promising strategy for UC prevention.

Aucubin (Auc) is an iridoid glycoside widely distributed in medicinal plants, including *Eucommia ulmoides* , *Plantago asiatica* , and *Aucuba japonica* (Kartini et al. 2023; Pan et al. 2025). Over the past decades, Auc has been extensively investigated as a multifunctional natural scaffold (Arafat et al. 2026; Zeng et al. 2020), and accumulating evidence has established a broad spectrum of pharmacological applications, including anti‐inflammatory, antioxidant, hepatoprotective, anti‐fibrotic, neuroprotective, and osteoprotective activities (Bi et al. 2025; Mu et al. 2025), as well as anticancer effects accompanied by regulation of the gut microbiota (Shao et al. 2022). More recently, Auc has attracted growing attention in intestinal biology: it alleviates DSS‐induced colitis by suppressing inflammatory pathways, restoring intestinal barrier integrity, and rebalancing the gut microbiota (Zhang, Qiao, et al. 2025), and its protective effects have been demonstrated to be microbiota‐dependent, being partly mediated by the microbiota–SCFAs–GPR41/GPR43 axis (Zhang, Ge, et al. 2026). Nevertheless, a systematic investigation of whether Auc prevents UC by reshaping the gut microbiota and its metabolic profile is still lacking.

In this study, we employed a dextran sulfate sodium (DSS)‐induced acute colitis mouse model to evaluate whether Auc confers protective effects against colitic injury and intestinal barrier damage. By integrating proteomics, 16S rRNA sequencing, and untargeted metabolomics, we aimed to elucidate the mechanisms of Au, focusing on host protein expression, gut microbiota composition, and metabolite variations. Our results demonstrate that Auc fortifies the intestinal barrier by restructuring the gut microecology and activating the tryptophan–indole metabolic pathway, providing a theoretical basis for its potential application in UC prevention.

## 2 Materials and Methods

### 2.1 Animals, Reagents, and Experimental Design

Six‐week‐old male specific pathogen‐free (SPF) C57BL/6 mice were obtained from Henan Skbth Biotechnology Co. Ltd. (SCXK (Yu) 2025–0005) and housed in the Experimental Animal Center of Changzhi Medical College. The animal experimental protocol (DW2026005) was reviewed and approved by the Animal Ethics Committee of Changzhi Medical College. Aucubin (purity ≥ 98% by HPLC) was obtained from Chengdu Aijicui Biotechnology Co. Ltd. 5‐aminosalicylic acid (5‐ASA; analytical reagent grade, purity ≥ 99%; catalog No. S30083‐25 g) was purchased from Shanghai Yuanye Bio‐Technology Co. Ltd. (Shanghai, China).

After 1 week of acclimatization, 40 C57BL/6 mice were randomly divided into five groups (Figure 1A), each with eight mice: normal control (NC), model (DSS), positive control (5‐ASA, 200 mg/kg), Aucubin low‐dose (Auc‐L, 50 mg/kg), and Aucubin high‐dose (Auc‐H, 100 mg/kg). The aucubin doses of 50 and 100 mg/kg were selected based on the effective dose range reported in previous animal studies. Specifically, aucubin doses of 40, 60, and 80 mg/kg have been reported to exert therapeutic effects in experimental colitis models (Zhang, Zhan, et al. 2025), while 50 mg/kg showed beneficial effects in an osteoarthritis model (Wang et al. 2019). Therefore, 50 mg/kg was selected as the lower dose because it is within the previously reported effective range, whereas 100 mg/kg was selected as a higher dose to further evaluate whether aucubin exerts dose‐dependent protective effects. Mice in the Aucubin groups were pre‐treated by intragastric gavage for 14 consecutive days. From Day 15 (i.e., after the gavage period), mice in all groups except the NC group were given free access to 2.5% (w/v) DSS in drinking water for 7 days to induce acute ulcerative colitis. The severity of colitis was evaluated daily using the disease activity index (DAI), which was calculated as the sum of three subscores: body weight loss, fecal trait, and fecal blood. Body weight loss was scored as 0 (no change), 1 (1% < loss ≤ 5%), 2 (5% < loss ≤ 10%), 3 (10% < loss ≤ 15%), or 4 (> 15%); fecal trait was scored as 0 (normal), 1 (soft but still formed), 2 (loose stool), 3 (very soft), or 4 (diarrhea); and fecal blood was scored as 0 (normal), 2 (hemoccult‐positive), or 4 (severe bleeding). The three subscores were summed to yield a total DAI score ranging from 0 to 12, with higher scores indicating more severe colitis (Ji et al. 2025). Body weight change was calculated relative to the body weight recorded immediately before DSS administration (day 14). Samples were collected at the experimental endpoint for subsequent analysis. Eight mice were initially assigned to each group. Samples for histological staining (*n* = 3), Enzyme‐Linked Immunosorbent Assay (ELISA) (*n* = 5), proteomic analysis (*n* = 3), metabolomic profiling (*n* = 6), and 16S rRNA sequencing (*n* = 6) were randomly selected from the same experimental cohort. The specific sample size for each assay was determined according to sample availability, assay requirements, and predefined quality‐control criteria.

**FIGURE 1:**
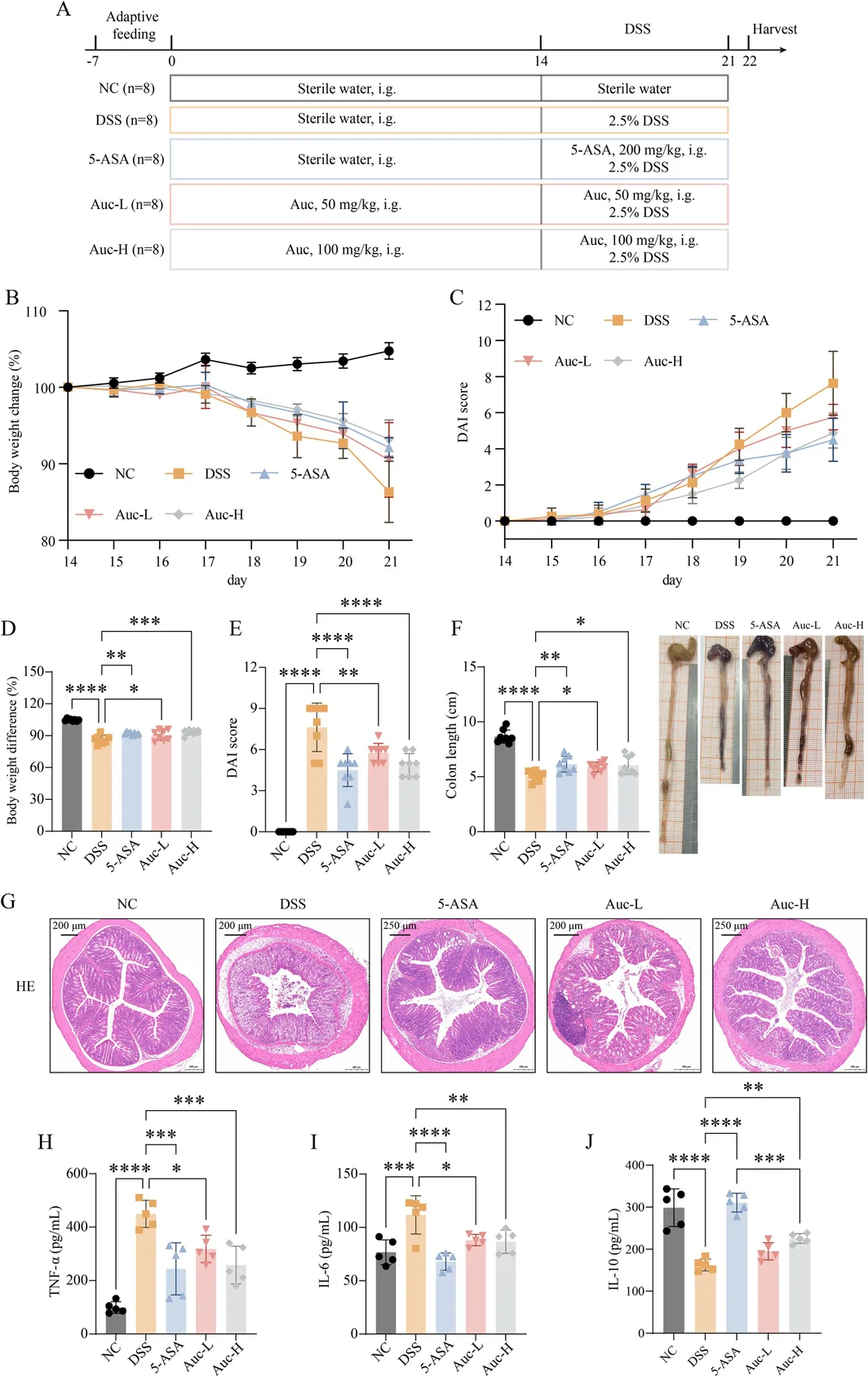
Effects of Auc on clinical symptoms and colonic pathological damage in DSS‐induced UC mice. (A) Schematic diagram of the experimental design. (B) Body weight change curves. (C) DAI score curves. (D, E) Percentage of body weight change (D) and DAI scores (E) of mice in each group at the experimental endpoint. (F) Representative photographs of colons and quantitative analysis of colon length. (G) Representative H&E staining images of colonic tissues. (H–J) ELISA results of serum TNF‐α (H), IL‐6 (I), and IL‐10 (J) levels. (*n* = 3–8). *p < 0.05, **p < 0.01, ***p < 0.001, ****p < 0.0001.

### 2.2 Histopathological Staining

Distal colon tissues were collected from each group and fixed in 4% paraformaldehyde for at least 24 h. Paraffin sections (4–5 μm thick) were prepared using standard procedures. Hematoxylin and eosin (H&E) staining was then performed to evaluate colonic tissue structure, crypt integrity, and inflammatory cell infiltration. Alcian Blue‐Periodic Acid‐Schiff (AB‐PAS) staining was used to identify mucopolysaccharides and mucins. All slides were scanned using a Saiviewer scanner (LG‐FS80, Wuhan Saiviewer) and analyzed using Saiviewer image analysis software.

### 2.3 Immunohistochemistry and Immunofluorescence

For immunohistochemistry (IHC), we used an anti‐mucin 2 (Muc2) primary antibody (GB11344, Servicebio, Wuhan, China). After antigen retrieval, blocking, and incubation with a secondary antibody, mucin proteins were visualized using DAB chromogen and counterstained with hematoxylin.

For immunofluorescence (IF) analysis, colon sections were dewaxed and subjected to antigen retrieval. The sections were then incubated overnight with primary antibodies against zonula occludens‐1 (ZO‐1, GB15195, Servicebio, Wuhan, China) and Occludin (GB15149, Servicebio, Wuhan, China). Fluorescently labeled secondary antibodies were then applied, and nuclei were counterstained with DAPI. The sections were scanned and analyzed using Saiviewer software (Servicebio, Wuhan, China); the IF module was used to quantify the distribution of tight junction proteins and count positively stained cells.

### 2.4 Enzyme‐Linked Immunosorbent Assay (ELISA)

At the end of the experiment, blood samples were collected and centrifuged to obtain serum. Serum levels of TNF‐α, IL‐6, IL‐10, and lipopolysaccharide (LPS) were then measured using ELISA kits.

### 2.5 Astral DIA Quantitative Proteomics

Total protein was extracted from colon tissues and quantified. After trypsin digestion, peptides were quantified using a Pierce kit. Peptides were separated on a Vanquish Neo UHPLC system and detected with an Orbitrap Astral mass spectrometer operating in DIA mode. Mass spectrometry data were processed using DIA‐NN v1.8.1 software in library‐free mode. After normalization and quantification using the MaxLFQ algorithm, differentially expressed proteins (DEPs) were screened using *t*‐tests or ANOVA (|log_2_FC| ≥ 0.585 and *p* ≤ 0.05). Subsequent bioinformatic analyses included GO, KEGG, and KOG functional annotation and enrichment analysis, combined with protein domain annotation, subcellular localization, and signal peptide prediction.

### 2.6 16S rRNA Microbiome Sequencing

Total microbial DNA was extracted from cecal contents. The V3‐V4 hypervariable region of the 16S rRNA gene was amplified by PCR, and sequencing libraries were constructed. Paired‐end sequencing was performed on the NovaSeq 6000 platform. Denoising was performed using the Deblur algorithm to generate amplicon sequence variants (ASVs). Species annotation was performed using Mothur software against the SILVA database, providing community composition information at the phylum, class, order, family, genus, and species levels. Alpha and beta diversity metrics were used to evaluate the microbial community structure. In addition, LEfSe analysis (LDA score > 4) was employed to identify microbial biomarkers that differed significantly between groups.

### 2.7 Untargeted Metabolomics Analysis

Mouse cecal contents were thawed and ground in liquid nitrogen. A 20 mg powder sample was mixed with 70% methanol–water extraction solution containing internal standards, and the supernatant was collected for LC–MS/MS analysis. Metabolites were filtered based on QC samples with CV < 0.3 and a comprehensive score > 0.5. After log_2_ transformation and zero‐centering, differential metabolites were screened using an OPLS‐DA model (200 permutation tests) with criteria of |log_2_FC| ≥ 1, VIP > 1, and *p* < 0.05. To explore the biological significance, KEGG and HMDB databases were used for functional annotation and enrichment analysis. In addition, MSEA enrichment analysis and ROC curve analysis were performed.

### 2.8 Statistical Analysis

Data are expressed as mean ± SD. Statistical analysis and graphical representation were performed using GraphPad Prism software (version 9.5.0, GraphPad Software, San Diego, CA, USA). For multiple‐group comparisons, one‐way ANOVA followed by Dunnett's or Tukey's multiple comparison test was used. *p* < 0.05 was considered statistically significant.

## 3 Results

### 3.1 Auc Alleviates Symptoms of DSS‐Induced Ulcerative Colitis in Mice

We evaluated the protective effects of Auc by establishing a DSS‐induced acute UC model (Figure 1A). Compared with the NC group, DSS induction caused a marked decline in body weight (Figure 1B,D), a sustained elevation in the DAI (Figure 1C,E), and significant colon shortening (Figure 1F) (*p* < 0.001). However, pre‐treatment with Auc (50 mg/kg and 100 mg/kg) significantly attenuated these pathological changes, evidenced by attenuated weight loss, reduced DAI scores, and attenuation of colon shortening. Notably, the Auc‐H group exhibited comparable improvements to the 5‐ASA positive control group in terms of body weight loss, DAI scores, and colon length, with no statistically significant differences between these two groups (*p* > 0.05).

The protective effect of Auc was further corroborated by H&E staining: whereas the DSS group displayed marked mucosal damage, crypt loss, and extensive inflammatory cell infiltration, Auc treatment notably preserved intestinal morphology and reduced inflammatory infiltration (Figure 1G). Biochemical analyses further demonstrated that both low and high doses of Auc significantly reduced the overproduction of the pro‐inflammatory cytokines TNF‐α and IL‐6 (Figure 1H,I). In contrast, a significant increase in the anti‐inflammatory cytokine IL‐10 was observed only in the high‐dose Aucubin group (100 mg/kg; Figure 1J); however, this increase was significantly lower than that observed in the 5‐ASA group (*p* < 0.001). Collectively, these findings indicate that Auc exerts a marked preventive and protective effect against DSS‐induced UC by suppressing inflammatory responses and reducing pathological damage.

### 3.2 Auc Alleviates DSS‐Induced Intestinal Barrier Dysfunction

To further investigate the effects of Auc on the intestinal barrier, we examined the integrity of the mucus and physical barriers in the mouse colon. As shown by AB‐PAS staining (Figure 2A) and IHC (Figure 2B), the NC group exhibited abundant goblet cells and mucus production. In contrast, DSS treatment significantly decreased goblet cell numbers, thinned the mucus layer, and reduced Muc2 expression. Auc intervention, particularly in the Auc‐H group, significantly alleviated these changes, increasing goblet cell numbers and enhancing Muc2 secretion. Regarding the physical barrier, IF analysis demonstrated that the fluorescence intensity of the tight junction proteins ZO‐1 and Occludin was significantly reduced and discontinuously distributed in the colonic mucosa of the DSS group, indicating compromised barrier integrity (Figure 2C–E). In the Auc‐treated groups, the expression of these tight junction proteins was upregulated in a dose‐dependent manner, restoring the morphology of intercellular tight junctions. In addition, serum LPS levels were abnormally elevated in the DSS group, indicating increased intestinal permeability (Figure 2F). Auc intervention effectively reduced serum LPS levels (*p* < 0.001), further confirming that Auc repairs DSS‐induced barrier damage and reduces gut‐derived endotoxin translocation by maintaining mucus secretion and strengthening tight junctions.

**FIGURE 2:**
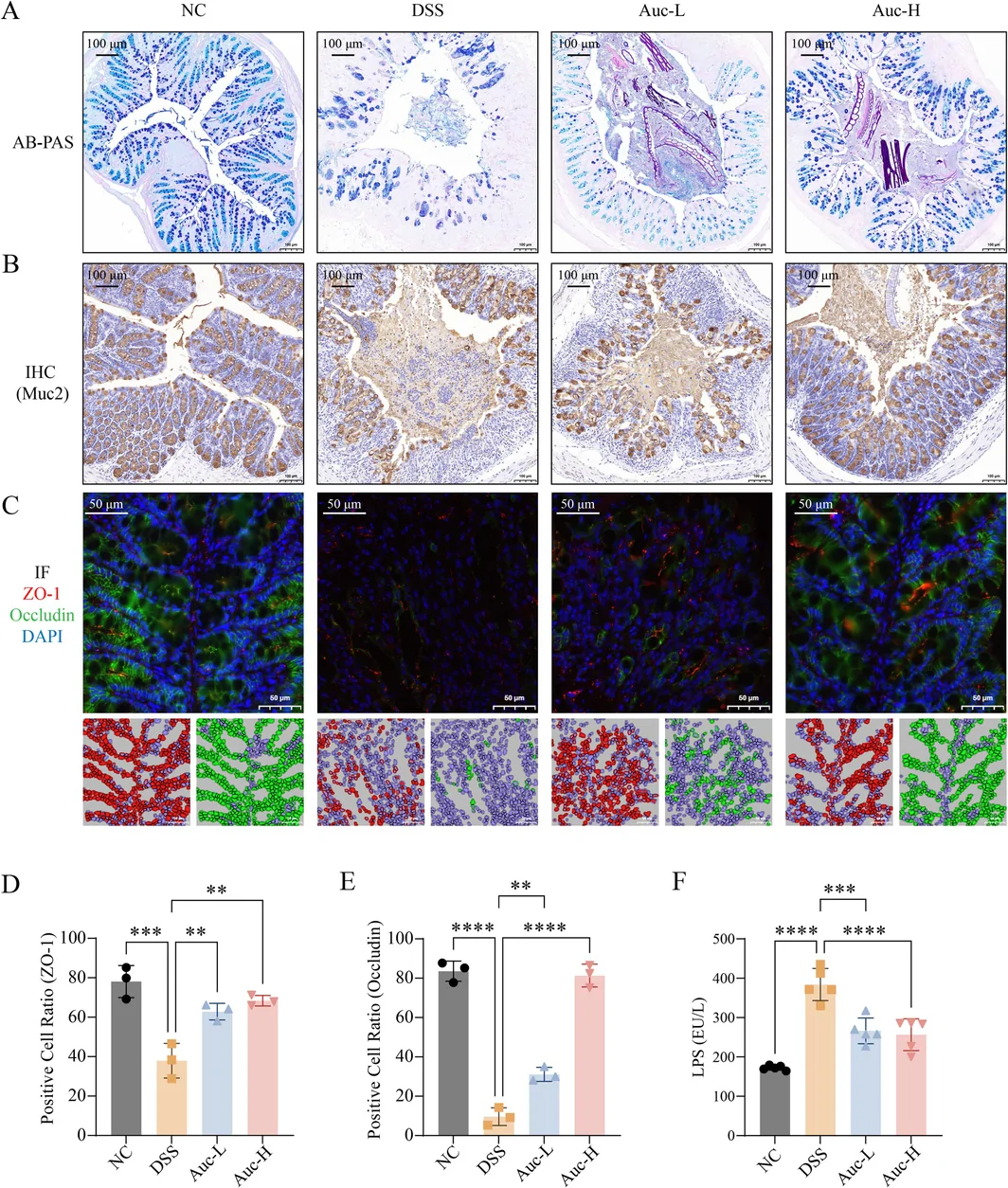
Auc alleviates DSS‐induced intestinal barrier dysfunction. (A) AB‐PAS staining results of colonic tissues. (B) Expression of Muc2 protein in colonic tissues detected by IHC. (C) Distribution and expression of tight junction proteins ZO‐1 (red) and Occludin (green) in colonic tissues detected by IF. Nuclei were stained with DAPI (blue). (D, E) Quantitative statistical analysis of the proportion of ZO‐1 (D) and Occludin (E) positive cells in colonic tissues. (F) Detection results of serum LPS levels. (*n* = 3–5). **p < 0.01, ***p < 0.001, ****p < 0.0001.

### 3.3 Proteomic Analysis of the Molecular Mechanism of Auc Protection Against DSS‐Induced Colitis

To understand the molecular mechanisms underlying the effects of Auc on UC, we performed proteomic analysis of colon tissues. A total of 11,978 proteins were identified, of which 11,708 were quantified (Figure 3A). Using a cutoff of |log_2_FC| ≥ 0.585 and *p* < 0.05, 2180 proteins were significantly altered in the DSS group compared with the NC group (1557 upregulated and 623 downregulated). After Auc‐H treatment, the expression of 679 proteins was significantly modulated (186 upregulated, 493 downregulated) (Figure 3B). In addition, hierarchical clustering heatmap analysis confirmed that Auc‐H treatment restored the colonic protein expression profile to a state resembling that of the NC group (Figure 3C). Subcellular localization analysis showed that the DEPs modulated by Auc were predominantly located in the extracellular space, nucleus, and cytoplasm (Figure 3D). Protein domain enrichment analysis revealed that the DEPs were primarily associated with immunoglobulin V‐set domains and serine protease inhibitors (Figure 3E). Furthermore, KEGG pathway enrichment analysis indicated that the effects of Auc‐H were mainly attributable to the regulation of immune‐ and inflammation‐related pathways, including the complement and coagulation cascades, the NOD‐like receptor signaling pathway, and neutrophil extracellular trap formation (Figure 3F). A circular heatmap of selected DEPs (Figure 3G) showed that Auc effectively modulated the expression of key proteins, including complement factors, defensins, and inflammation‐related chemokines.

**FIGURE 3:**
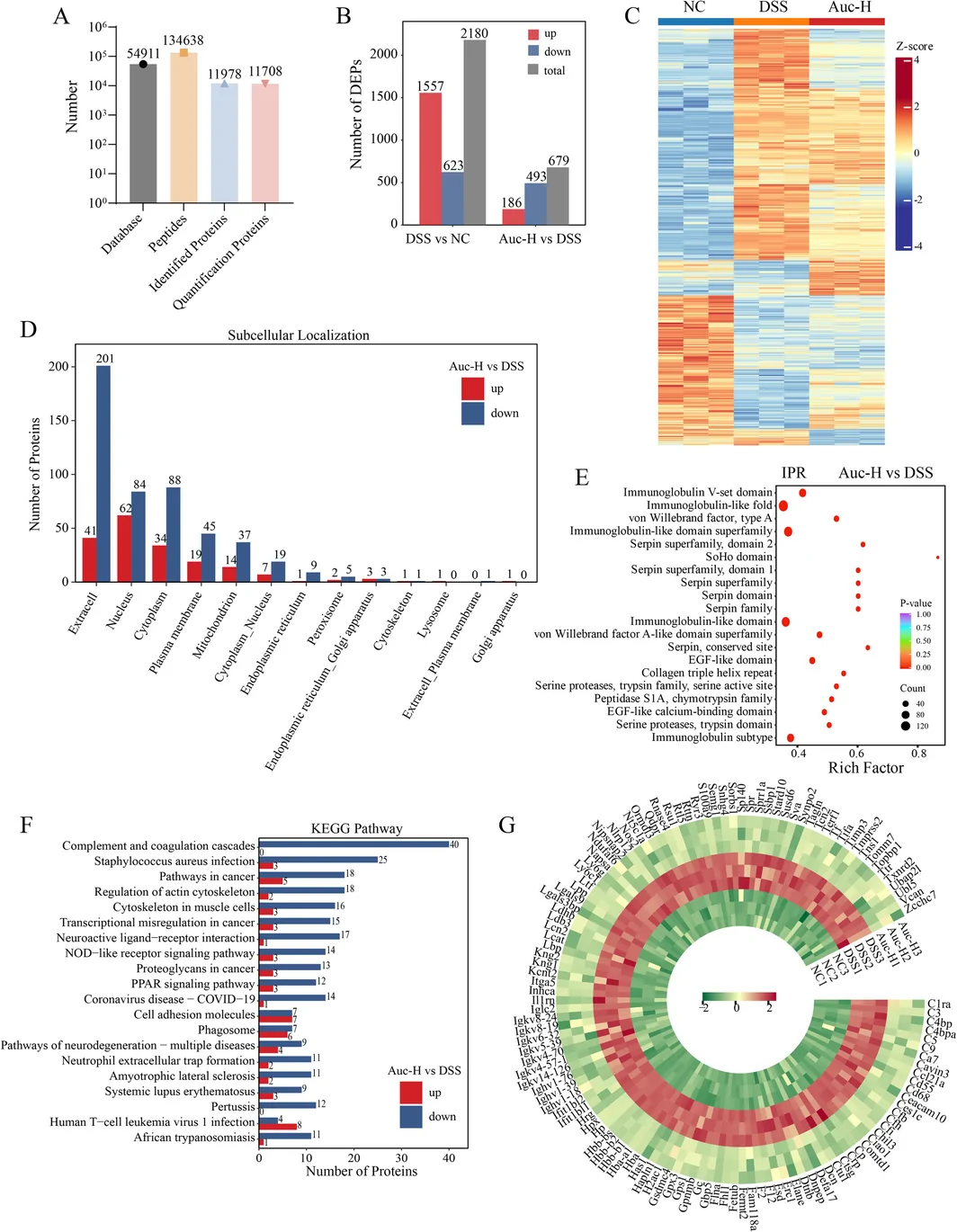
Proteomics‐based analysis of the molecular mechanisms by which Auc protects against DSS‐induced colitis. (A) Overview of proteomics identification. (B) Statistical bar chart of the number of DEPs in comparison groups (DSS vs. NC and Auc‐H vs. DSS) (|log_2_FC| ≥ 0.585 and *p* ≤ 0.05). (C) Hierarchical clustering heatmap based on DEPs. (D) Subcellular localization distribution of DEPs in the Auc‐H vs. DSS comparison group. (E) Bubble chart of InterPro (IPR) protein domain enrichment analysis. (F) Bar chart of KEGG signaling pathway enrichment. (G) Circular heatmap of differentially expressed proteins among groups. (*n* = 3).

### 3.4 Auc Modulates Gut Microbiota Dysbiosis in DSS‐Induced Colitis Mice

Gut microbiota dysbiosis plays a critical role in the pathogenesis of UC. We therefore investigated the regulatory effects of Auc on the gut microbiota by 16S rRNA sequencing. The Venn diagram (Figure 4A) revealed that 432 ASVs were common to the NC, DSS, and Auc‐H groups, with the Auc‐H group exhibiting 13 unique ASVs. Alpha diversity analysis revealed that DSS induction significantly diminished the Shannon index, whereas Auc intervention did not significantly elevate diversity levels (Figure 4B). Principal coordinates analysis (PCoA) based on Bray‐Curtis distances (Figure 4C) revealed a clear difference in microbial composition between the DSS and NC groups. However, Auc‐H samples tended to cluster closer to the NC group along the first principal coordinate. Regarding community structure (Figure 4D,E), Auc‐H treatment relatively increased Bacteroidota and decreased Firmicutes abundance, consequently altering the Firmicutes/Bacteroidota (F/B) ratio (Figure 4F). LEfSe analysis (Figure 4G) further identified taxa characteristic of each group, with greater abundance of *Akkermansia muciniphila* and *Prevotellaceae* in the DSS group, whereas the Auc‐H group showed significantly increased abundance of Firmicutes genera, including *Lachnospiraceae* and *Clostridiales*. In addition, single‐species abundance analysis (Figure 4H,I) confirmed that Auc‐H significantly increased the abundance of *g_unidentified_Lachnospiraceae* and *s_Clostridiales_bacterium*_CIEAF_020. These findings suggest that Auc can rectify DSS‐induced dysbiosis by promoting the abundance of potentially beneficial bacteria, such as butyrate producers.

**FIGURE 4:**
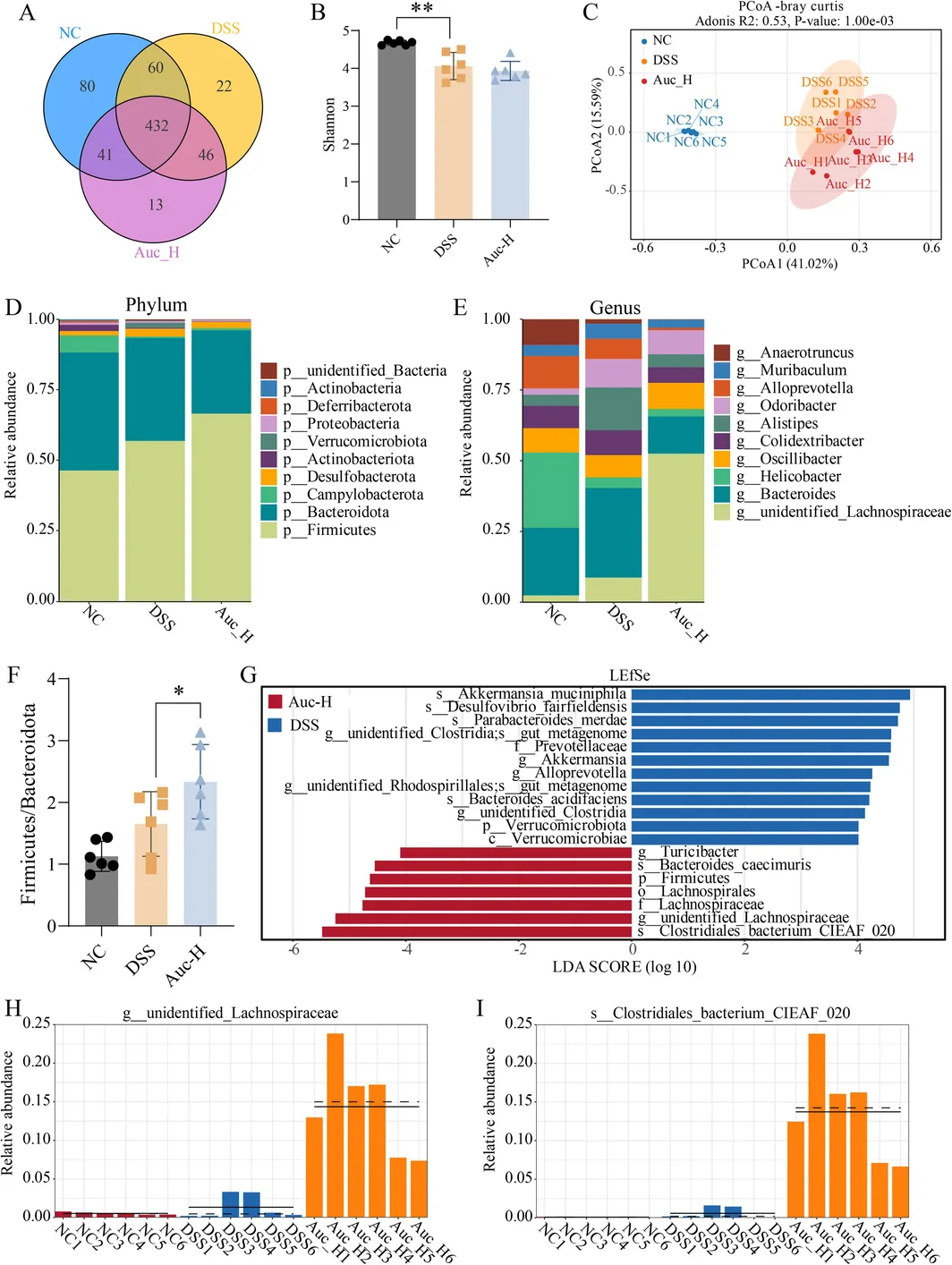
Regulatory effects of Auc on the gut microbiota structure of DSS‐induced UC mice. (A) Venn diagram based on ASV counts. (B) Shannon index. (C) PCoA analysis based on Bray‐Curtis distances. (D, E) Relative abundance composition of gut microbiota at the phylum (D) and genus (E) levels. (F) Statistical analysis of the Firmicutes/Bacteroidota ratio in the colon. (G) Horizontal bar chart of differential taxa based on LEfSe analysis (LDA > 4). (H, I) Relative abundance distribution of the key differential genus g_unidentified_Lachnospiraceae (H) and species s_Clostridiales_bacterium_CIEAF_020 (I) across samples. (*n* = 6). *p < 0.05, **p < 0.01.

### 3.5 Auc Reshapes the Intestinal Metabolic Profile of DSS‐Induced Colitis Mice

To investigate the effects of Auc on the intestinal metabolic environment, we performed untargeted metabolomic analysis of cecal contents. Using thresholds of |log_2_FC| ≥ 1, *p* < 0.05, and VIP ≥ 1, volcano plots (Figure 5A,B) revealed 330 differential metabolites between the DSS and NC groups (218 upregulated and 112 downregulated). Auc‐H intervention significantly restored the levels of 120 of these metabolites (86 upregulated, 34 downregulated). PCA (Figure 5C) revealed distinct clustering of the NC, DSS, and Auc‐H groups, indicating that DSS induction caused marked metabolic disturbance, whereas Auc‐H treatment significantly altered the metabolic profile. Hierarchical clustering heatmap (Figure 5D) and K‐means clustering analysis (Figure 5E) demonstrated that Auc‐H significantly reversed the DSS‐induced downregulation trend of metabolites (Subclass 4). Within Subclass 4, Auc‐H notably restored the levels of metabolites associated with anti‐inflammatory and barrier‐repairing functions, including tyrosol, xanthurenic acid, mesalazine, and 1‐methylnicotinamide (Figure 5F). In addition, Auc‐H significantly elevated the levels of several tryptophan derivatives and indole metabolites, including N‐acetyl‐D‐glucosamine, 5‐hydroxyindoleacetic acid (5‐HIAA), 5‐hydroxyindole (5‐HI), indole‐3‐carbinol (I3C), and indole‐3‐ethanol (Figure 5G) (*p* < 0.01). These findings suggest that the protective effect of Auc against UC may be attributable to its modulation of the gut metabolite profile, particularly the activation of the tryptophan‐indole metabolic pathway.

**FIGURE 5:**
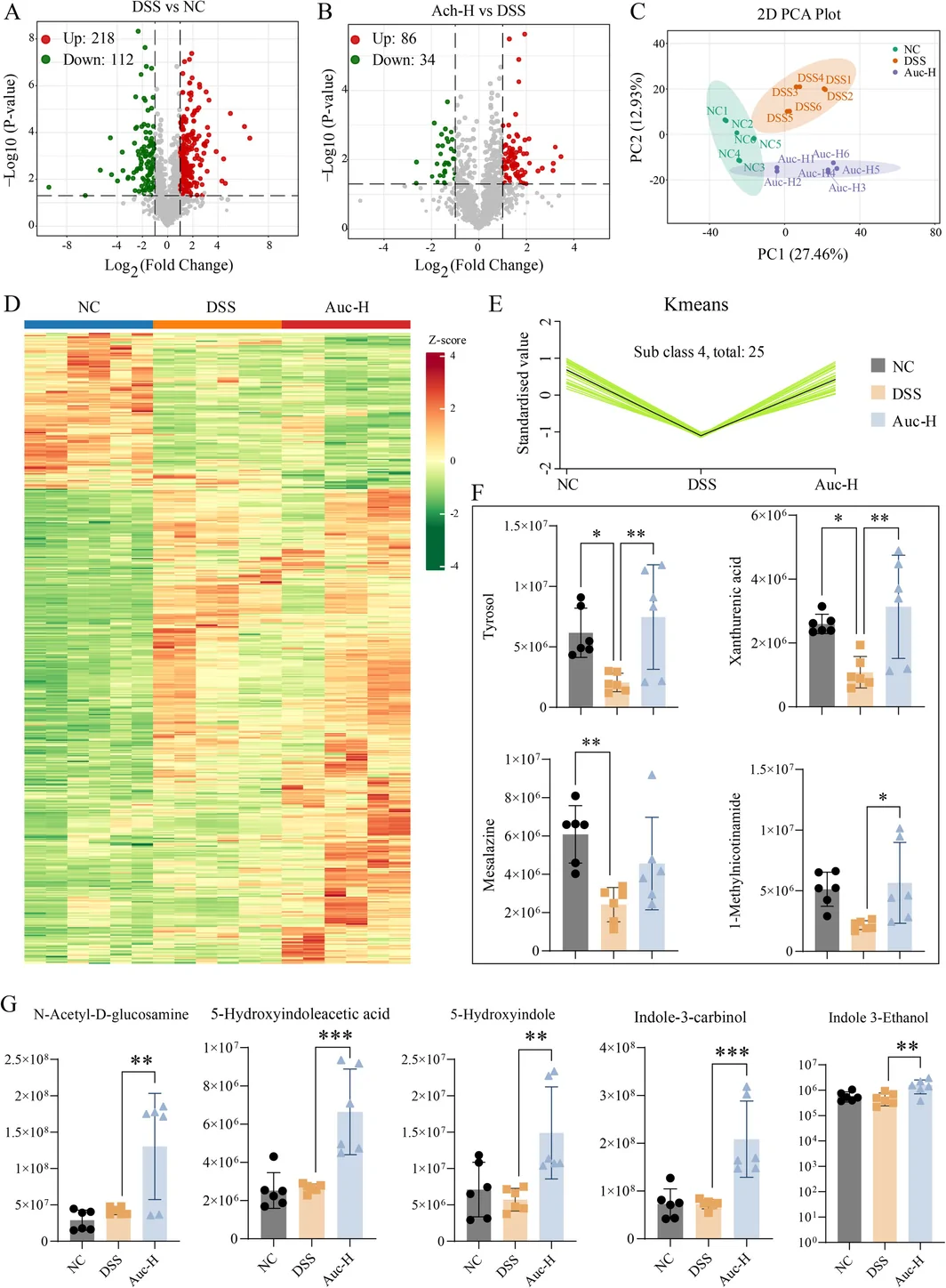
Effects of Auc on the metabolic profile of cecal contents in DSS‐induced colitis mice. (A, B) Volcano plots of differential metabolites (|log_2_FC| ≥ 1, VIP > 1, and *p* < 0.05). (C) PCA analysis. (D) Hierarchical clustering heatmap of differential metabolites. (E) K‐means clustering analysis plot highlighting the metabolite cluster (Subclass 4) restored by Auc‐H intervention. (F) Relative abundance statistics of key differential metabolites in Subclass 4 (Tyrosol, Xanthurenic acid, Mesalazine, 1‐Methylnicotinamide). (G) Relative abundance statistics of N‐Acetyl‐D‐glucosamine, 5‐HIAA, 5‐HI, Indole‐3‐carbinol, and Indole 3‐Ethanol. (*n* = 6). *p < 0.05, **p < 0.01, ***p < 0.001.

### 3.6 Spearman Correlation Analysis

To further explore the association between Auc‐induced changes in gut bacteria and metabolic profiles, we performed Spearman correlation analysis. This analysis was performed on differentially abundant bacterial genera and key differential metabolites (Figure 6A). The results showed that beneficial bacteria significantly increased by Auc, such as *Lachnospiraceae* and *Clostridiales_bacterium_CIEAF_020*, were positively correlated with various gut‐protective metabolites (*p* < 0.01). These metabolites mainly included indole derivatives (e.g., 5‐hydroxyindole, indole‐3‐carbinol, indole‐3‐ethanol), tyrosol, and xanthurenic acid. Furthermore, correlation network analysis (Figure 6B) provided insight into the complex relationships among these elements. Indole metabolites and the previously identified functional bacteria occupied central positions in the network, showing high connectivity. Collectively, these findings suggest that Auc exerts anti‐inflammatory and gut barrier‐protective effects, likely by regulating the gut microbiome to promote beneficial bacteria that produce metabolites such as indoles.

**FIGURE 6:**
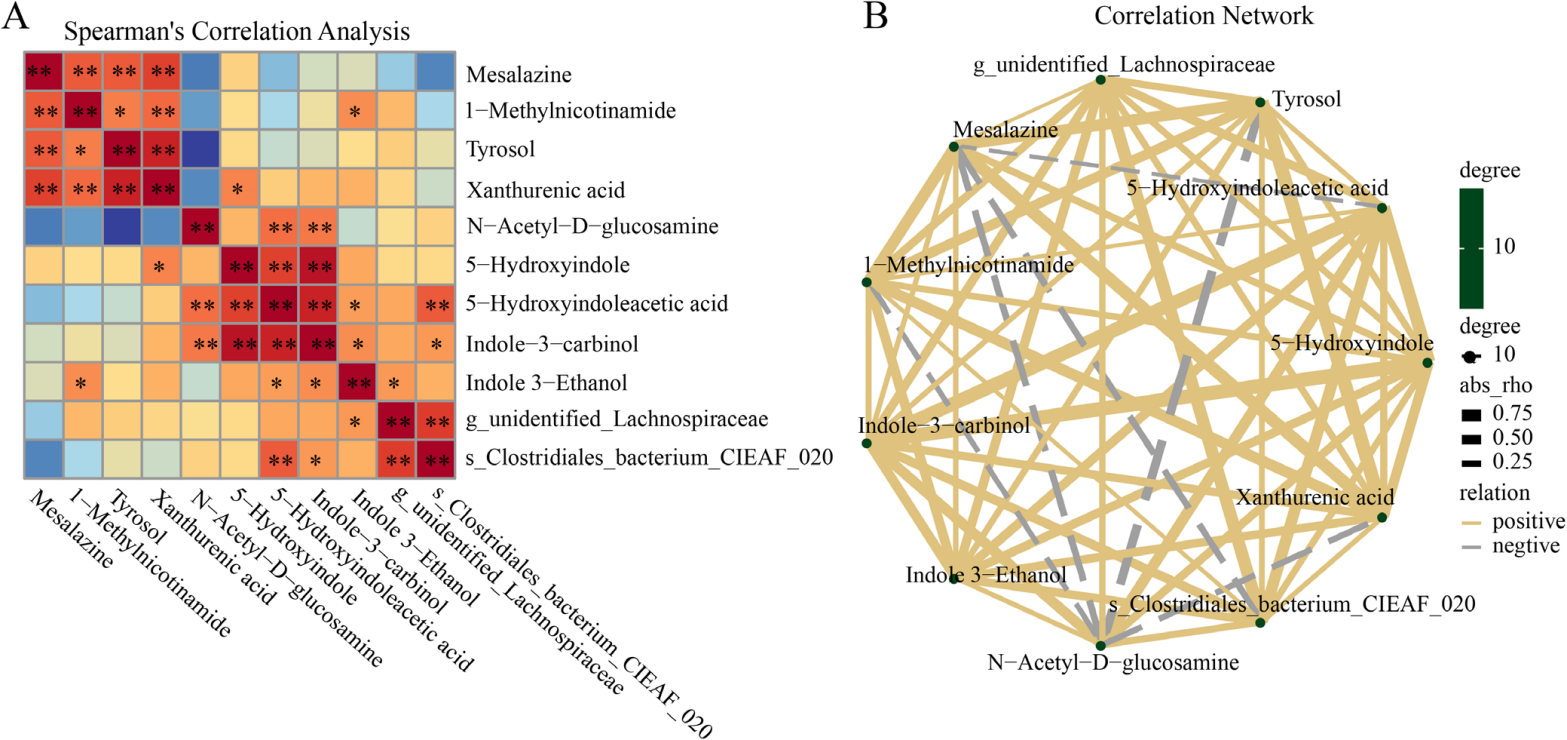
Correlation analysis between differential gut microbiota and differential metabolites. (A) Spearman correlation heatmap between characteristic bacterial genera and key differential metabolites. (B) Correlation network diagram between gut microbiota and metabolites. *p < 0.05, **p < 0.01.

## 4 Discussion

UC is a complex inflammatory bowel disease characterized by intestinal immune dysregulation, disruption of the epithelial barrier, and gut microbiota imbalance. This study systematically evaluated the protective effects of Auc against DSS‐induced UC in mice. The findings demonstrate that Auc pre‐treatment substantially alleviates the clinical manifestations of colitis, including weight loss, elevated DAI scores, and colon shortening, and confers multifaceted protection against colonic damage by modulating the colonic proteome, restructuring the gut microbiota, and enhancing the metabolic microenvironment. Aucubin is a major iridoid glycoside constituent of medicinal plants such as *Eucommia ulmoides* and *Plantago asiatica* . A 2025 study using high‐performance thin‐layer chromatography (HPTLC)–densitometry showed that aucubin content reached 16.75% in ultrasonically assisted extracts obtained from non‐leaf parts of *Eucommia ulmoides* , including the bark and root bark, further indicating that *Eucommia ulmoides* ‐derived extracts can provide relatively abundant aucubin (Amalia et al. 2026). The doses of 50 and 100 mg/kg used in this study were selected based on previous pharmacological studies and are consistent with the dosing ranges potentially achievable using plant‐derived extracts or formulations, thereby supporting the potential translational feasibility of aucubin as a candidate for dietary or pharmacological intervention.

Auc contributes to the restoration of the primary gut protective barrier by strengthening its physical and chemical defenses. Breakdown of the intestinal epithelial barrier is an early event in the development of UC, allowing bacterial endotoxins to enter the bloodstream and trigger systemic inflammation. In this study, Auc significantly increased the expression of the tight junction proteins ZO‐1 and Occludin and restored goblet cell numbers and Muc2 levels. These results are consistent with recent studies showing that natural compounds can activate endogenous mucosal repair processes (Lu et al. 2025; Zhang, Yang, et al. 2026). The finding that Auc reduces serum LPS levels supports the notion that its protective effect is mediated by regulating intestinal permeability, thereby breaking the vicious cycle of leaky gut‐driven inflammation. Regarding the serum cytokine profile, TNF‐α and IL‐6 are pivotal pro‐inflammatory mediators that drive epithelial apoptosis, neutrophil recruitment, and mucosal injury in UC, whereas IL‐10 is a key anti‐inflammatory cytokine that restrains excessive immune activation; the concurrent suppression of TNF‐α and IL‐6 and elevation of IL‐10 observed here indicate that Aucubin rebalances the inflammatory response rather than merely suppressing it. In addition, IL‐22—an epithelium‐repairing cytokine downstream of AhR activation—was not quantified in the present study; given its central role in mucosal healing and its close mechanistic link to the indole–AhR axis discussed below, direct measurement of IL‐22 warrants inclusion in future experimental designs.

Remodeling of the gut microbiota and metabolites represents a core mechanism through which Auc exerts its pharmacological effects. The 16S rRNA sequencing data revealed that Auc substantially increased the Firmicutes‐to‐Bacteroidetes (F/B) ratio, with a particularly increased relative abundance of *Lachnospiraceae* and *Clostridiales*. *Lachnospiraceae* is a well‐established beneficial bacterial family in the gut microbiota, known for its capacity to ferment dietary fiber, thereby producing short‐chain fatty acids and contributing to tryptophan metabolism (Huang et al. 2023; Li et al. 2024). Contrary to expectations, the model group exhibited higher abundance of *Akkermansia muciniphila* (AKK) than the control group. This finding appears to contradict the well‐documented beneficial roles of AKK in enhancing mucus secretion, repairing tight junctions, and regulating immunity (Gu et al. 2021; Hua et al. 2025). This seemingly paradoxical phenomenon may stem from the pathological mechanism by which DSS destroys the intestinal mucus layer, providing an abnormally rich substrate for AKK, a mucin‐degrading bacterium, and thereby stimulating its compensatory proliferation. Thus, the elevated abundance of AKK in this context is more likely a pathological marker of severe gut dysbiosis than a sign of health. Recent studies have revealed the context‐dependent, double‐edged nature of AKK: in inflammatory environments with immune dysregulation and barrier collapse, its overgrowth and metabolites may aggravate infection or immune disorder (Wang, Li, Lei, et al. 2025). Accordingly, our findings underscore that the significance of AKK should not be assessed solely on the basis of its abundance, but requires holistic interpretation in the context of the host's immune status and barrier integrity.

Correlation analyses demonstrated a robust positive association between Lachnospiraceae and tryptophan metabolites, such as I3C, 5‐HI, and 5‐HIAA. Indole compounds, mainly produced by gut microbial transformation of tryptophan, act as natural activators of AhR (Chen et al. 2024). AhR activation has been shown to increase IL‐22 release, which promotes mucosal barrier repair and antimicrobial peptide production (Sun et al. 2020; Wu, Xu, et al. 2026). In addition, the increased intestinal xanthurenic acid levels after Auc treatment have been shown to alleviate colitis by activating the AhR pathway (Wu, Wei, et al. 2025). Furthermore, the elevated levels of mesalazine, tyrosol, and 1‐methylnicotinamide induced by Auc should not be overlooked. Mesalazine is itself a first‐line drug for mild‐to‐moderate UC. Tyrosol, a potent antioxidant metabolite, has been shown to alleviate DSS‐induced UC by scavenging reactive oxygen species and inhibiting NF‐κB signaling activation (Yu et al. 2024). Moreover, 1‐methylnicotinamide, an active metabolite of nicotinamide, shows significant potential in cardiovascular protection, anti‐inflammatory effects, and slowing of aging processes (Yin et al. 2025). A study by Hou et al. demonstrated that 1‐methylnicotinamide attenuated inflammation and modulated the microbiota in necrotizing enterocolitis (Wu, Wei, et al. 2025). Therefore, Auc likely acts as a microecological regulator that indirectly activates gut immune regulation and repair mechanisms by promoting the growth of indole‐producing probiotics and the production of anti‐inflammatory metabolites.

Finally, proteomic analysis revealed the molecular mechanisms underlying the protective effects of Auc. This study found that Auc significantly reversed the DSS‐induced proteomic alterations. Functional enrichment analysis using InterProScan (IPR) revealed that Auc predominantly affected domains associated with immunoglobulins, serpins, and von Willebrand factor. This finding is consistent with the KEGG pathway enrichment results. In particular, Auc markedly suppressed pro‐inflammatory pathways, including the complement and coagulation cascades, the NOD‐like receptor signaling pathway, and neutrophil extracellular trap formation. Of note, the complement system and NOD‐like receptors are essential components of innate immunity, and their excessive activation contributes to persistent exacerbation of intestinal mucosal inflammatory injury (Chen et al. 2025; Wang, Li, Xu, et al. 2025). The downregulation of these pathways by Auc supports its regulatory effects on the gut microbiota and metabolites. We propose that by elevating intestinal indole metabolites to activate AhR, Auc may further inhibit NOD‐like receptor‐mediated inflammasome activation in colonic tissue, thereby reducing aberrant neutrophil infiltration. In addition, complement cascade inhibition by Auc may help mitigate vascular endothelial injury and mucosal bleeding, consistent with the observed improvement in bloody stools. With respect to the individual parameters examined, the protective effects of Aucubin can be mechanistically integrated as follows: the attenuation of colon shortening and histopathological injury reflects the preservation of epithelial integrity; the restoration of Muc2, ZO‐1, and Occludin indicates reinforcement of both the mucus and tight‐junction barriers; the proteomic changes, dominated by the complement and coagulation cascades and NOD‐like receptor signaling, reveal suppression of innate immune overactivation; and the 16S rRNA and metabolomic alterations demonstrate reprogramming of the microbiota–metabolite axis toward indole–AhR signaling. Regarding the positive control, 5‐ASA primarily acts through direct inhibition of epithelial COX/PGE2 and NF‐κB signaling, whereas Aucubin appears to act predominantly through microbiota–metabolite reprogramming and barrier reinforcement. Thus, while both agents ultimately dampen colonic inflammation, their primary mechanisms are distinct and potentially complementary.

While this investigation corroborates the beneficial preventative impact of Auc on UC, certain constraints are acknowledged. Initially, despite multi‐omics data indicating Auc's modulation of the intestinal barrier through the microbiota‐metabolite axis, the lack of germ‐free or antibiotic‐treated mouse models precludes a conclusive assertion that Auc's protective effect is solely reliant on the gut microbiota. Furthermore, while proteomic analyses enabled the identification of protein targets, this investigation did not utilize gene‐knockout mouse models to confirm the precise signaling pathways implicated. The main focus of this research was on Aucubin's ability to prevent disease. More studies are needed to determine its effectiveness in treating colitis after it has started. In addition, several other limitations should be acknowledged: only male mice were used, and therefore sex‐specific differences in the response to Aucubin could not be evaluated; and because ulcerative colitis is frequently associated with oxidative stress, redox status parameters (such as MDA and H_2_O_2_ levels, as well as enzymatic and non‐enzymatic antioxidants) were not evaluated in this study, and a comprehensive assessment of colonic redox status should be included in future work.

In summary, this study demonstrates that Aucubin is an effective preventive agent for ulcerative colitis. Its mechanism involves not only direct anti‐inflammatory and barrier‐repairing effects but, more importantly, systemic remodeling of the gut microbiota‐metabolite axis. In particular, it activates endogenous repair programs by promoting the enrichment of microbiota associated with indole metabolite production. With regard to future applications, we propose that Aucubin is more likely to be translated through a standardized pharmaceutical formulation or a concentrated botanical extract with a defined Aucubin content, rather than through direct consumption of raw plant materials, as the latter cannot guarantee reproducible dosing; alternatively, Aucubin‐enriched functional foods may serve as a practical preventive strategy for at‐risk populations.

## Author Contributions

**Yanghui Bi:** conceptualization, methodology, writing – original draft, funding acquisition. **Pengyu Chen:** methodology, investigation. **Shuya Jia:** methodology, investigation. **Shiyan Jia:** methodology, investigation. **Zhanhao Lian:** methodology, investigation. **Yuanfang Zheng:** methodology, validation. **Jiabin Guo:** methodology, validation. **Jianxia Xue:** methodology, validation. **Sinan Cheng:** conceptualization, writing – review and editing, supervision, funding acquisition.

## Funding

This work was supported by Scientific and Technologial Innovation Programs of Higher Education Institutions in Shanxi (No. 2022L359); Technology Commission Foundation of Shanxi Province (No. 202203021222315); Scientific Research Project of Health Commission of Shanxi Province (No. 2023115); Shanxi Province Fundamental Research Project (202303021222275), the Shanxi Province Higher Education Technology Innovation Project (2023L198) and Doctor research startup foundation of Changzhi Medical College (BS202502).

## Conflicts of Interest

The authors declare no conflicts of interest.

## Data Availability

The data that support the findings of this study are available on request from the corresponding author. The data are not publicly available due to privacy or ethical restrictions.
